# Low tidal volume ventilation with low PEEP during surgery may induce lung inflammation

**DOI:** 10.1186/s12871-016-0209-y

**Published:** 2016-07-30

**Authors:** Hitoshi Sato, Kyota Nakamura, Yasuko Baba, Shoko Terada, Takahisa Goto, Kiyoyasu Kurahashi

**Affiliations:** 1 Departments of Anesthesiology and Critical Care Medicine, Yokohama City University Graduate School of Medicine, 3-9 Fukuura, Kanazawaku, Yokohama, Japan; 2 Present address: Advanced Critical Care and Emergency Center, Yokohama City University Medical Center, Kanagawa, Japan; 3 Present address: Operation Department, Yokohama City University Medical Center, Kanagawa, Japan; 4 Present address: Department of Anesthesiology, Yokohama City University Medical Center, Kanagawa, Japan; 5 Present address: Division of Critical Care Medicine, Yokohama City University Medical Center, Kanagawa, Japan

**Keywords:** Cytokines, Hepatectomy, Systemic Inflammatory Response Syndrome, Ventilator-Induced Lung Injury, Micro-sampling Method

## Abstract

**Background:**

Compared to conventional tidal volume ventilation, low tidal-volume ventilation reduces mortality in cased of acute respiratory distress syndrome. The aim of the present study is to determine whether low tidal-volume ventilation reduces the production of inflammatory mediators in the lungs and improves physiological status during hepatic surgery.

**Methods:**

We randomly assigned patients undergoing hepatectomy into 2 groups: conventional tidal-volume vs. low tidal-volume (12 vs. 6 mL•kg^−1^ ideal body weight) ventilation with a positive end-expiratory pressure of 3 cm H_2_O. Arterial blood and airway epithelial lining fluid were sampled immediately after intubation and every 3 h thereafter.

**Results:**

Twenty-five patients were analyzed. No significant changes were found in hemodynamics or acid–base status during the study. Interleukin-8 was significantly elevated in epithelial lining fluid from the low tidal-volume group. Oxygenation evaluated immediately after admission to the post-surgical care unit was significantly worse in the low tidal-volume group.

**Conclusions:**

Low tidal-volume ventilation with low positive end-expiratory pressure may lead to pulmonary inflammation during major surgery such as hepatectomy.

**Trial registration:**

The effect of ventilatory tidal volume on lung injury during hepatectomy that requires transient liver blood flow interruption. UMIN000021371 (03/07/2016); retrospectively registered

## Background

In acute respiratory distress syndrome (ARDS), ventilation with a low tidal volume (V_T_) reduces mortality compared to a conventional V_T_ [1, 2]. Recent studies have shown that ventilation with a conventional tidal volume is also associated with sustained cytokine production in the lungs in patients without lung injury at the onset of mechanical ventilation [3–6]. Furthermore, incidences of lung injury have been reported after major surgery in those without any pre-existing lung diseases [7]. In those studies [3–6], the protective ventilation strategy consists of low V_T_ ventilation, relatively high positive end-expiratory pressure (PEEP), and lung recruitment maneuver. During hepatectomy, however, surgeons require low PEEP to reduce bleeding from cut surface of the liver [8]. Our question was: when high PEEP, one part of lung protective approaches, is unavailable, does the low tidal volume ventilation strategy have utility? To answer the question, we proposed a study that aimed to evaluate the effect of low tidal volume ventilation during surgery under the condition with a restricted PEEP level (3 cmH_2_O).

We conducted a prospective, randomized controlled study on patients undergoing hepatic surgery under two different V_T_ ventilation conditions assigned randomly to determine whether low V_T_ ventilation reduces lung injury and improves lung physiology during hepatic surgeries. The primary outcome of the present study was the change in pro-inflammatory cytokine concentrations in the lungs. Secondary outcomes were oxygenation during and immediately after the surgery and the duration of hospital stay after the surgery. We hypothesized that (a) proinflammatory mediators increase in the circulation after hepatic surgery with the Pringle maneuver that causes a temporal hepatic blood flow interruption; (b) airway inflammation is induced when a conventional V_T_ is used during surgery; and (c) compared to conventional V_T_ ventilation, low V_T_ ventilation during hepatectomy reduces airway inflammation and prevents lung injury under a condition of a limited PEEP.

## Methods

### General protocol and patients

This prospective, randomized, controlled study was performed at Yokohama City University Hospital. The data were collected from October 2008 to September 2009, with approval from the institutional review board (Date of IRB approval: 08-01-2007; approval number: 07-021), and written informed consent was obtained from all the patients preoperatively.

Patients aged between 20 and 85 years, undergoing hepatectomy, were considered eligible for enrolment in this study. Patients with an American Society of Anesthesiologists’ physical status (ASA-PS) value of 3 and above, pre-existing lung disease, tumor in the portal vein or inferior vena cava, requirement of bile duct or gastrointestinal tract repair, or requirement of additional surgical procedures other than hepatectomy were excluded.

Patients were randomly assigned to those ventilated with a V_T_ of 12 mL per predicted body weight (kg) (TV12) or with a V_T_ of 6 mL per predicted body weight (TV6). The assignment was performed using a random number table by an investigator who was not involved in data collection and was notified to anesthesiologists who were not involved in the study using an envelope method. The investigators who collected the data and samples were blinded to the ventilation settings at any time of the experiment. Mechanical ventilation was performed in a volume-controlled mode, with the ratio of the duration of inspiration to the duration of expiration (I/E) of 1:2 and an end-inspiratory pause time of 10 %, using an anesthesia machine (Drager Fabius GS, Drager Medical, Telford, PA, USA). The patients did not receive premedication. Propofol 2 mg•kg^−1^, vecuronium 0.1 mg•kg^−1^, and fentanyl 100 μg was administered to facilitate orotracheal intubation with a cuffed tube. General anesthesia was maintained with sevoflurane 0.6–1.5 % and was supplemented by epidural anesthesia with mepivacaine.

The target arterial partial oxygen pressure (PaO_2_) of approximately 150 mmHg was attained by adjusting the inspired oxygen fraction (F_I_O_2_) and the arterial partial carbon dioxide pressure (PaCO_2_) was maintained between 35 and 45 mmHg by changing the ventilation frequency referring to the previous blood gas analysis and end-tidal carbon dioxide pressure. PEEP was applied at 3 cm H_2_O in both the groups. Ephedrine was administered when the systolic blood pressure dropped below 80 mmHg. Methylprednisolone (8 mg•kg^−1^) was administered intravenously prior to the Pringle maneuver (obstruction of both branches of the hepatic artery and portal vein). Muscle relaxation was reversed with neostigmine and atropine when surgery was completed. Lungs were recruited manually with approximately 20 cmH_2_O for 15 to 20 s prior to extubation in both groups.

### Blood sampling and blood gas analysis

Arterial blood was drawn just prior to bronchoscopic microsampling (BMS), and blood gas analysis (BGA) was performed (model 860, Chiron Diagnostics, Emeryville CA, USA) every 3 h thereafter. Whole blood was centrifuged at 4 °C at 3000 RPM, and the plasma was aliquoted and stored at −80 °C until use. When the patient arrived in the post-anesthetic care unit (PACU), BGA was repeated.

### Bronchoscopic microsampling method

#### Epithelial lining fluid sampling

Epithelial lining fluid (ELF) was collected with BMS probes using a previously reported method [9]. Briefly, a BMS probe was inserted into the channel of a fibreoptic bronchoscope that was inserted into the tracheal tube. The tip of the BMS probe was attached to a segmental bronchus of the right middle lobe under optical guidance of a bronchoscope for 20 s. The BMS probe was then withdrawn from the bronchoscope. These procedures were repeated 3 times using 3 different BMS probes. The tips of the BMS probes, made of cotton, were inserted into pre-weighed test tubes. The tubes were sealed, weighed again with the probe tips, and stored at −80 °C. The collections were performed immediately after intubation and after 3 and 6 h.

#### Determination of the sample weight and ELF extraction

One milliliter of distilled water was added to each test tube containing BMS probes. The tubes were centrifuged at 4 °C for 10 min, and the supernatant was collected and aliquoted. The BMS probes were dried on a bench top at room temperature for 3 days and weighed. The weight of the collected sample was calculated using the following formula:

S  = (T + P_1_) – T – P_2_, where S is the sample weight, T is the weight of the tube, (T + P_1_) is the weight of the tube and the BMS probes after sampling, and P_2_ is the weight of the dried probes after extraction. A sample dilution factor (DF) in distilled water was then calculated as follows:

$$ \mathrm{D}\mathrm{F} = \left(\mathrm{S} + 1000\right)\ /\ \mathrm{S},\ \mathrm{where}\ \mathrm{S}\ \mathrm{is}\ \mathrm{the}\ \mathrm{sample}\ \mathrm{weight}\ \mathrm{in}\ \mathrm{milligrams}. $$

### Measurements of mediator concentrations in the blood and ELF

Cytokines and adhesion molecules were measured using an enzyme-linked immunosorbent assay (ELISA). Tumor necrosis factor (TNF)-α (Quantikine^®^ Human TNF-α/TNFSF1A, R&D Systems, Minneapolis, MN, USA), interleukin (IL)-8 (EH2IL8, Thermo Scientific, Rockford, IL, USA), and intercellular adhesion molecule (ICAM)-1 (EH5400, Thermo Scientific) levels were measured according to the manufacturer’s instructions.

### Clinical data collection

Preoperative data were collected from routine clinical documentation that was stored in the institutional medical record system. Intraoperative physiological and ventilatory data were recorded in a data sheet.

### Statistical analysis

All data were statistically analyzed using Statcel 2^nd^ edition (OMS Publishing, Tokorozawa, Japan). The student *t*-test and Mann–Whitney *U* test were used to assess quantitative variables. Variables measured only once were compared using an unpaired *t*-test. Variables that were measured repeatedly were compared using two-way repeated measures analysis of variance (ANOVA) followed by Bonferroni post hoc. Results were expressed as mean ± standard deviation; *p* < 0.05 was considered significant.

## Results

### Patient demography

**Fig. 1:**
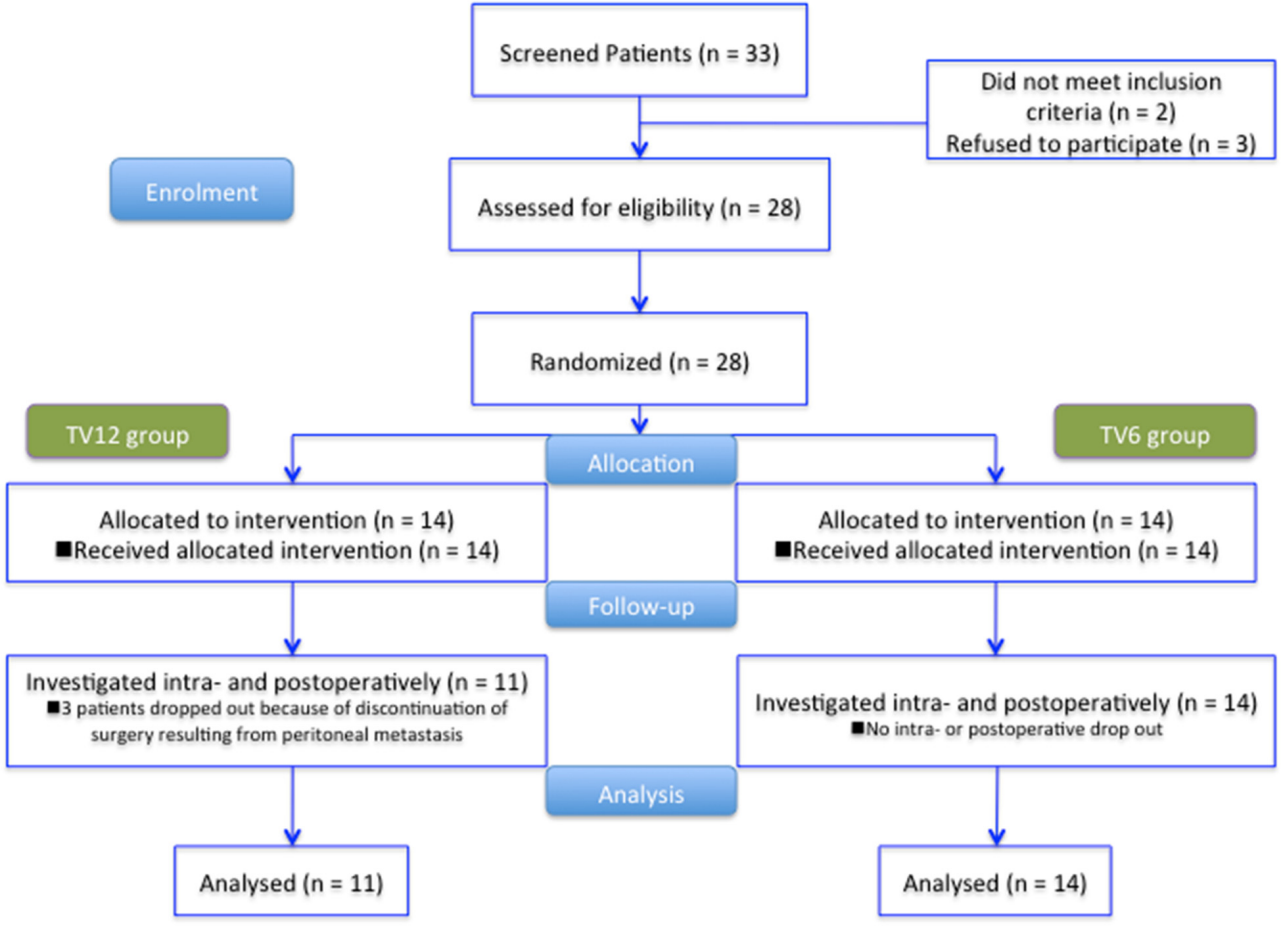
Consort flow diagram for the present study

A total of 28 patients were enrolled, and 14 patients were assigned to each group (Fig. 1). Three patients in the TV12 group were excluded because the operation was terminated before the completion of the study due to dissemination of tumor to the peritoneum. No differences were present in the demographic or clinical data between the groups (Table 1).

**Table 1:** Patient characteristics and the baseline P/F ratio

	TV 12	TV 6	*P*-value
Age,year^a^	69 (60/68)	63 (59/72)	0.3
Gender (male/female)^b^	8/3	10/4	0.94
Body weight,kg	57.5 ± 10.2	62.7 ± 10.7	0.25
Height,cm	164.5 ± 10.1	166.1 ± 6.9	0.66
Body mass index,kg•m^−2^	21.2 ± 2.7	22.6 ± 2.6	0.22
Operation time, min	512.8 ± 113.9	419 ± 199.3	0.23
Anesthesia time, min	606.1 ± 128.0	525.2 ± 207.3	0.32
Blood loss, mL	852.0 ± 465.0	852.0 ± 466.4	0.39
Baseline P/F ratio	501.6 ± 23.9	435.8 ± 27.6	0.09
Liver resection amount, %	38.5 ± 8.8	33.0 ± 17.4	0.38
Pringle maneuver, times^c^	3 (3/5.5)	4 (3/6)	0.75
Length of hospital stay after operation, days	12.5 ± 5.6	15.2 ± 9.0	0.38

Values are indicated as the mean ± SD otherwise indicated

Age ^a^is represented as the mean (range), gender ^b^as a number, and Pringle maneuver ^c^as median (25^th^ and 75^th^ percentiles)

### Physiological parameters

**Fig. 2:**
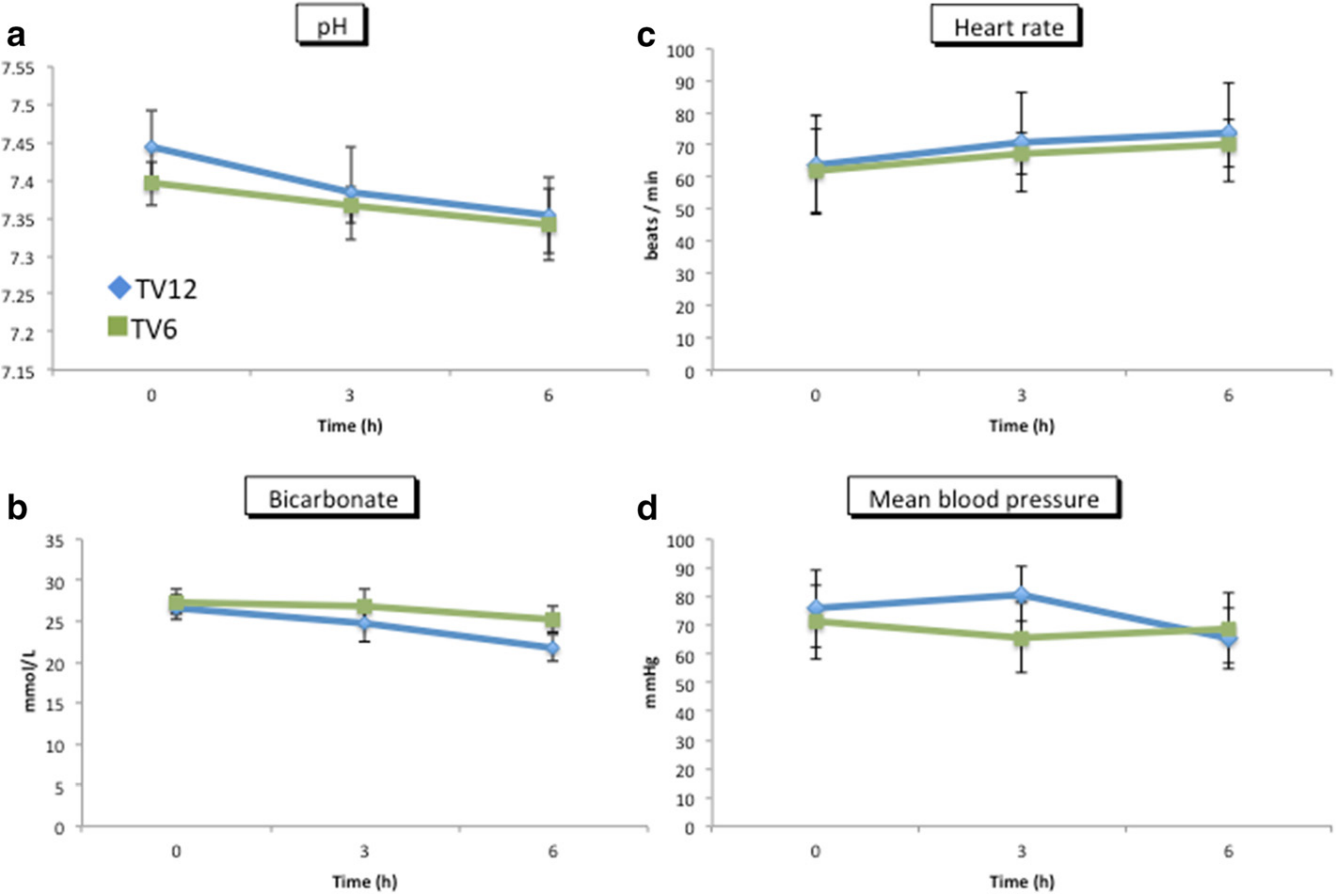
Changes in hemodynamics and BGA data. pH (**a**) and bicarbonate (**b**) were analyzed by blood gas analyses, whereas heart rate (**c**) and mean blood pressure (**d**) were obtained from a bedside monitor. Mean ± standard deviation. No significant differences were observed in BP, HR, or BGA between the groups

**Fig. 3:**
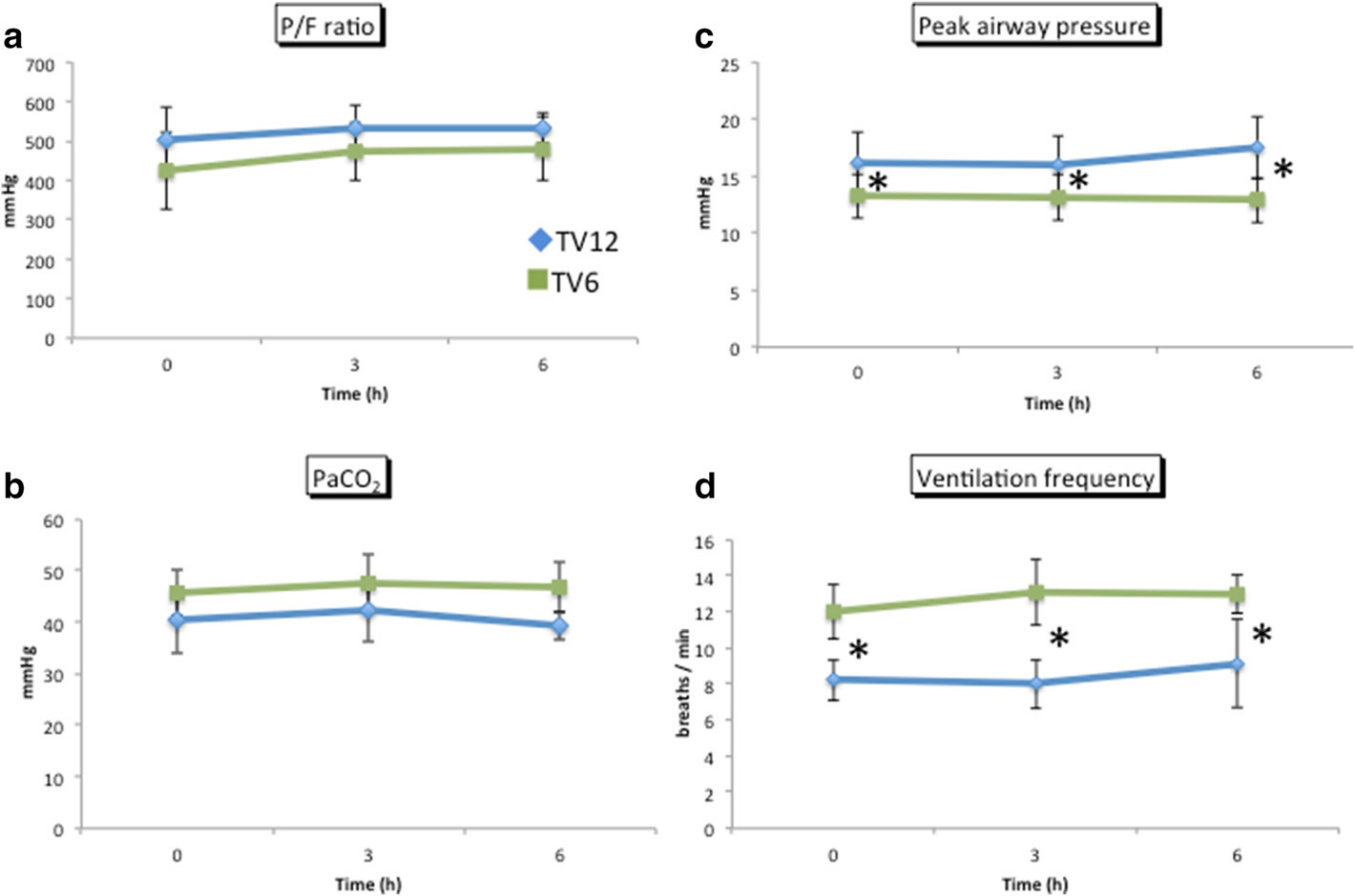
Changes in arterial blood gases, airway pressure, and ventilation frequency. **a** Changes in the P/F ratio were calculated by PaO_2_ analyzed by a blood gas analyzer and FIO_2_. **b** Changes in PaCO_2_ were analyzed by a blood gas analyzer. **c**, **d** Changes in peak airway pressure and ventilator frequency. Mean ± standard deviation. Peak airway pressure was significantly higher in the TV12 group. Ventilation frequency was greater in the TV6 group than in the TV12 group

**Fig. 4:**
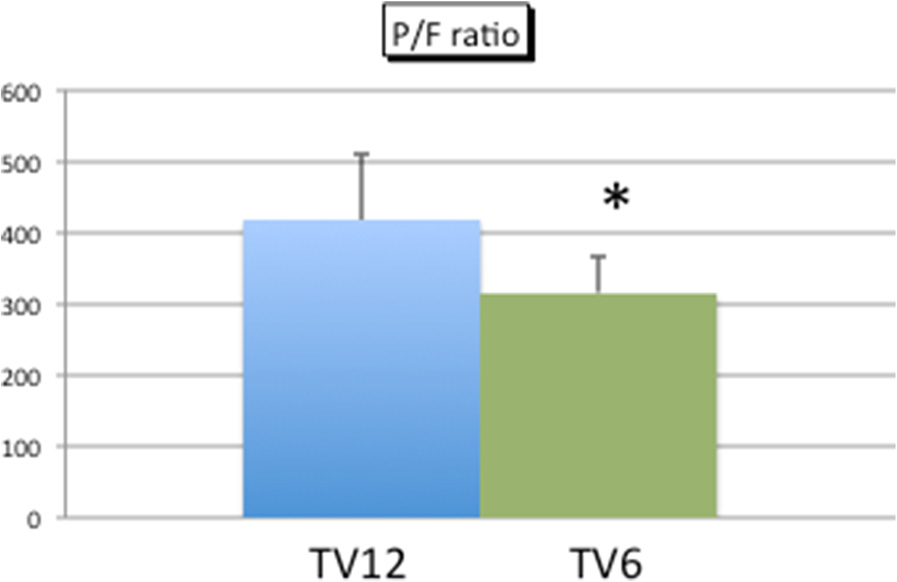
Postoperative P/F ratio in the PACU. Mean ± standard deviation. The P/F ratio evaluated just after admission to the PACU was higher in the TV12 group than in the TV6 group (*p* = 0.009)

No significant differences were found in pH, bicarbonate concentration, heart rate, or blood pressure between the groups (Fig. 2). The F_I_O_2_ was set between 0.3 and 0.5 in all patients. There was no significant difference in the P/F ratio (Fig. 3a) or the PaCO_2_ (Fig. 3b) between the two groups at any time point. Peak airway pressure was significantly higher in the TV12 group than in the TV6 group (Fig. 3c). To maintain the PaCO_2_ within the normal range, ventilation frequency was greater in the TV6 group than in the TV12 group (Fig. 3d). All patients were extubated in the operating room and were spontaneously breathing when they arrived at the PACU. The P/F ratio evaluated just after admission to the PACU was higher in the TV12 group than in the TV6 group (417 ± 92 versus 315 ± 49, *p* = 0.009) (Fig. 4).

### Biological parameters

**Fig. 5:**
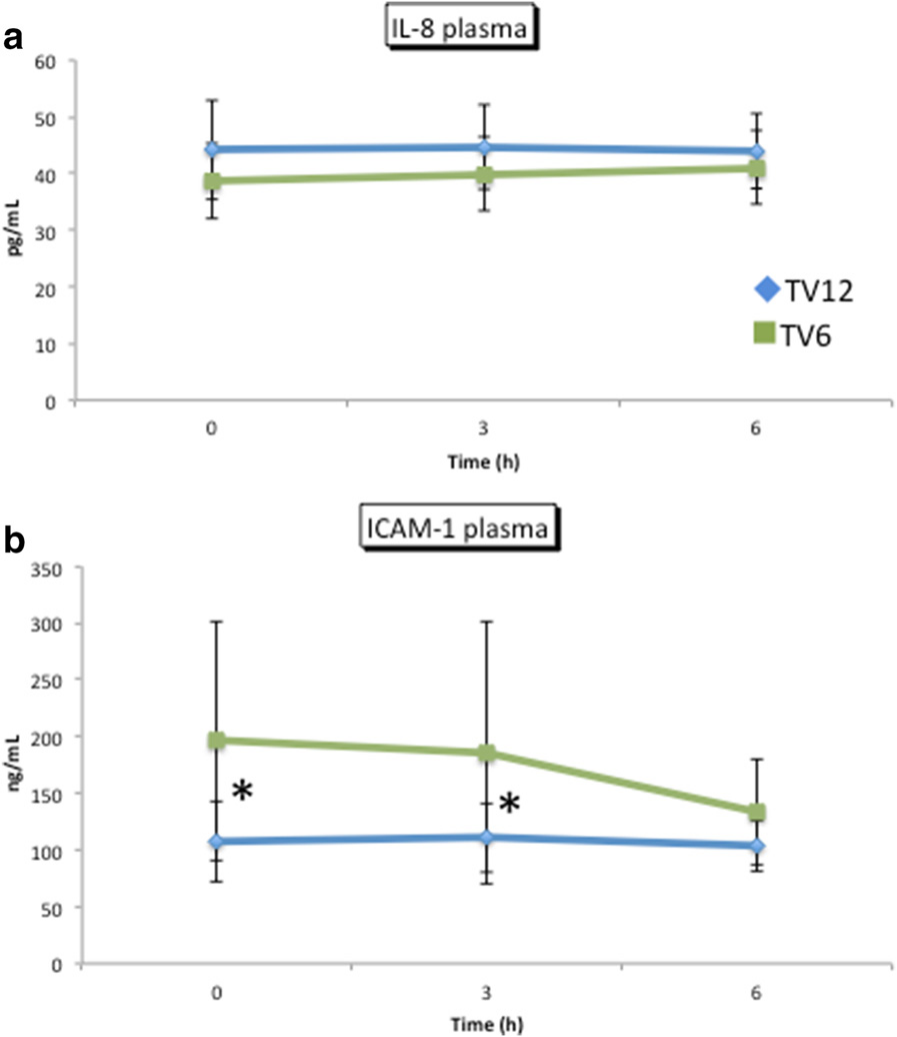
Plasma concentrations of IL-8 and ICAM-1. Mean ± standard deviation. No significant differences were observed in the plasma concentration of IL-8 between the groups (*p* = 0.17). Plasma ICAM-1 was significantly higher in the TV6 group than in the TV12 group (*p* = 0.03)

**Fig. 6:**
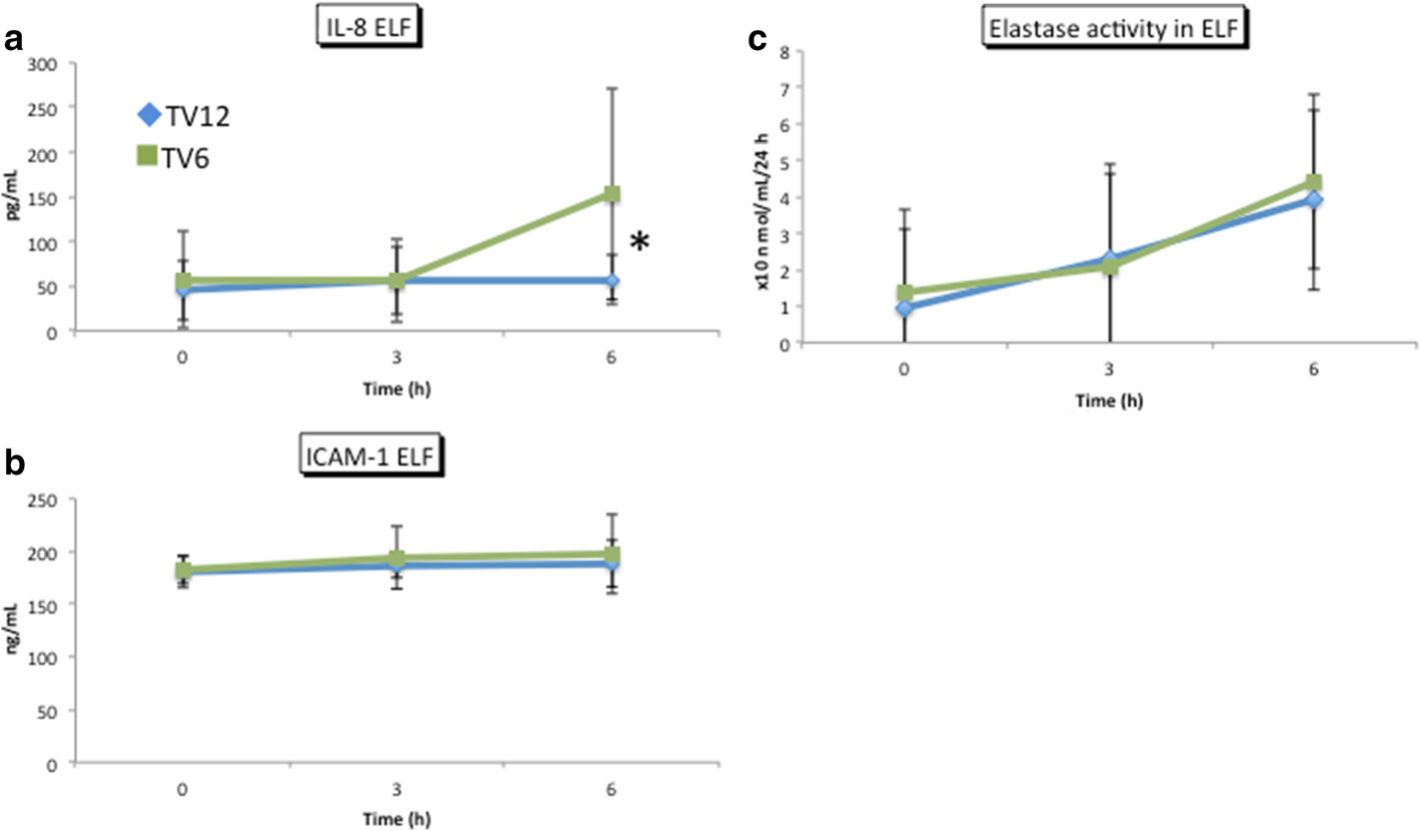
Concentrations of IL-8, ICAM-1, and elastase activity in the ELF. Mean ± standard deviation. The concentration of IL-8 in the ELF was significantly higher in the TV6 group than in the TV12 group (*p* = 0.04) and the post hoc analysis revealed a significant difference at 6 h (*p* = 0.03). No significant difference was observed in ICAM-1 (*p* = 0.31) or elastase activity (*p* = 0.7) in the ELF

No significant difference was found in the plasma concentration of IL-8 (*p* = 0.17) (Fig. 5a). TNF-α was below the detection limit (1.6 pg•mL^−1^) in the plasma and ELF samples obtained from all the patients. Elastase activity in the plasma was minimal in both the groups. ICAM-1 in the plasma was significantly higher in the TV6 group than in the TV12 group (*p* = 0.03; Fig. 5b). The concentration of IL-8 in the ELF was significantly higher in the TV6 group than in the TV12 group at 6 h (*p* = 0.03) (Fig. 6a). No significant difference was found in ICAM-1 (*p* = 0.31) or elastase activity (*p* = 0.7) in the ELF between the groups (Figs. 6b, c).

### Sharing our data

Data supporting our findings are available upon request.

## Discussion

### Main findings

The main findings of this study are:

1. Low tidal-volume ventilation during hepatectomy induced an increase in the concentration of IL-8 in the ELF collected during hepatectomy.
2. Low tidal-volume ventilation during hepatectomy resulted in a lower P/F ratio after surgery.

These were contrary to our hypothesis that low V_T_ventilation would reduce lung inflammation and preserve physiological lung functions following major surgery, compared to conventional ventilation.

### The mechanism of lung injury

Our hypothesis was based on studies that showed the benefits of low V_T_ ventilation in ARDS patients [1]. A considerable number of ARDS cases originate from extra-pulmonary complications including pan-peritonitis, cholecystitis, multiple injury, and massive transfusion. Severe systemic inflammation is a common occurrence in these conditions. Ischemia-reperfusion of organs or other parts of the body are the leading causes of systemic inflammation. The liver is one of the largest organs in the human body; approximately 25 % of the entire blood flows into the liver. Therefore, repeated hepatic ischemia-reperfusion may be a major cause of systemic inflammation. Takeuchi and colleagues showed polymorphonuculear cell (PMN) recruitment in the lungs, proinflammatory cytokine elevation in the blood and lung homogenates, and pulmonary edema in mice after 90 min of liver ischemia and reperfusion [10]. Our previous study showed that lung injury occurs following repeated hepatic ischemia and reperfusion with high V_T_ ventilation in rats [11]. Taken together, hepatic surgeries performed with the Pringle maneuver is a potential leading cause of lung injury; therefore, reducing V_T_ during hepatectomy is a reasonable strategy to prevent lung injury.

### Protective ventilation during surgery

Recently, several studies have been conducted regarding V_T_ and lung functions during surgery. Michelet and colleagues showed that concentrations of IL-8, IL-6, and TNF-α in the plasma were lower in patients who underwent esophagectomy with lower V_T_ ventilation (5 mL•kg^−1^, PEEP 5 cm H_2_O) than in those who underwent esophagectomy with higher V_T_ ventilation (9 mL•kg^−1^, PEEP 0 cm H_2_O) during one-lung ventilation [12]. Wolthuis and colleagues showed that the concentration of IL-8 in broncho-alveolar lavage (BAL) fluid was significantly lower in patients ventilated with a low V_T_ (6 mL•kg^−1^, PEEP 10 cm H_2_O) than in those ventilated with a large V_T_ (12 mL•kg^−1^, PEEP 0 cm H_2_O) during elective surgery [13]. Severgnini and colleagues reported that low V_T_ ventilation (6–8 mL•kg^−1^, PEEP 6–8 cm H_2_O) during abdominal surgery improved postoperative pulmonary function and reduced the modified Clinical Pulmonary Infection Score as compared with a standard ventilation strategy (10–12 mL•kg^−1^, PEEP 0 cm H_2_O) [14]. A recent, randomized controlled trial showed that ventilation with a V_T_ of 6 to 8 mL per kg of predicted body weight with a PEEP of 6 to 8 cm of H_2_O and a recruitment maneuver reduced major pulmonary complications after abdominal surgery compared to ventilation with a V_T_ of 10–12 mL per kg of predicted body weight with no PEEP and no recruitment maneuver [15]. These studies reported that low V_T_ ventilation during surgery results in reduced inflammation or better lung functions after the surgery as compared with relatively higher V_T_ ventilation. We should note that relatively higher PEEP and/or lung recruitment maneuver were applied to the groups that are ventilated with lower V_T_ in those papers [12–15].

The results of the present study were in contrast to those of previous studies in terms of the correlation between the level of V_T_ and the post-surgical lung function. The most plausible reason for the discrepancy is the level of PEEP that was applied in the present study. We used relatively low PEEP (3 cmH_2_O) in both the groups, which may have influenced the results. Recently, there are a few papers that focused on the relationship between PEEP level during surgery and postoperative pulmonary complications in otherwise healthy patients. Ladha et al. retrieved anesthesia records and compared ventilation settings with respiratory complications [16]. Protective ventilation defined as a median PEEP of 5 cmH_2_O or more, a median tidal volume of less than 10 mL•kg^−1^ of predicted body weight, and a median plateau pressure of less than 30 cmH_2_O was associated with a decreased risk of postoperative respiratory complications. de Jong et al. retrieved anesthesia records and compared median PEEP of < 5 cmH_2_O, = 5 cmH_2_O, or > 5 cmH_2_O with respiratory outcome [17]. Application of PEEP > 5 cmH_2_O was associated with a significant lower odds of respiratory complications and decreased hospital length of stay in patients undergoing major abdominal surgery but not in patients undergoing craniotomy. These findings suggest that special consideration such as application of PEEP of 5 cmH_2_O or higher is necessary especially when abdominal surgery is undergone. More recently, a meta-analysis revealed that a protective lung ventilation, low V_T_ ventilation concomitant with PEEP and intermittent recruitment maneuver, showed a significant reduction in incidences of postoperative lung infection, atelectasis, acute lung injury, and length of hospital stay; whereas, low V_T_ alone failed to reduce some of the incidences [18]. In the present study, low V_T_ ventilation with low PEEP applied to patients undergoing hepatectomy failed to improve pulmonary function, which is consistent with the previous findings [16–18]. Moreover, it is important to understand that optimal V_T_ or PEEP for otherwise healthy patients undergoing surgery could be different from those for ARDS patients with a baby lung.

### Mechanism of deteriorated lung function

After 6 h of ventilation, we found that IL-8 in the ELF was higher in the TV6 group than in the TV12 group. Previous studies have shown an increase in IL-8 levels in atelectatic lungs. Lung collapse results in increased IL-8 levels in BAL fluid and the re-expansion of the lungs further increases IL-8 levels in rabbits [19]. One-lung ventilation resulted in an IL-8 increase in the ELF of the non-ventilated lungs [20]. These observations suggest that the increase in IL-8 levels in the ELF in the low V_T_ group in the present study was due to a repeated lung collapse and re-opening of the lungs (atelectrauma) during surgery due to low V_T_ ventilation concomitant with low PEEP. ICAM-1 in the plasma was significantly higher in the TV6 group than in the TV12 group. Plasma ICAM-1 is associated with poor clinical outcomes in patients with acute lung injury [21]. In that study, however, plasma ICAM-1 level is also elevated in the patients with hydrostatic pulmonary edema, who basically have minimal lung injury. In the present study, mean plasma ICAM-1 concentrations in the TV12 and TV6 were from 107 to 117 ng/mL and from 133 to 196 ng/mL, respectively. These values were identical to that for the patients with hydrostatic pulmonary edema in the previous study (median 177 ng/mL) [21], suggesting that the effects of plasma ICAM-1 in the present study on lung injury are minimal in both groups.

### Advantages of BMS method over BAL collection

Historically, BAL fluid has been used to assess the biochemical status of the airway; however, we collected bronchial ELF using the BMS method to assess lung inflammation in this study. There are a few advantages of BMS method over BAL collection. First, concerns have been raised related to patient safety during BAL collection, including desaturation during the procedure, surfactant breakdown, and a spreading of localized pathology. In fact, Bauer and colleagues showed a decrease in the PaO_2_/F_I_O_2_ ratio after BAL collection, regardless of the BAL volume used [22]. Second, it is not possible to quantitate the concentration of biomarkers in the airway because the exact dilution factor may not be obtained in this way. Lastly, it is inappropriate to obtain repeated BAL measurements within a short period of time because the biomarkers are washed out. In the BMS method, ELF is collected using an absorptive probe guided by a fiberoptic scope; thus, we were able to safely and repeatedly collect biochemical markers from the patients’ airways. In contrast to BAL, BMS has the following advantages when used to determine the biochemical status of the airway: oxygenation can be maintained during and after the procedure; alveolar surfactant is preserved; quantification of the biochemical markers is possible; and samples can be repeatedly obtained within a short duration [9].

### Limitations of the study

The present study has a few limitations. First, we did not find a relevant paper to refer to in terms of the standard deviations of the two groups and thus we did not perform power analysis. Accordingly, there may be type-two error in the results of the study. Second, a steroid was administered prior to the Pringle maneuver. It is mandatory to administer a steroid for hepatectomy at our institute, regardless of the study; however, this may have limited systemic inflammation in both of the groups. In fact, in both groups, TNF-α levels in the plasma were below the detection limit, and IL-8 levels in the plasma during the surgery were similar to that of baseline values. Third, in the previous paper, the median plasma sICAM-1 concentrations for survivor and non-survivor among patients with ARDS were 338 ng/mL and 737 ng/mL, respectively [21], whereas the plasma ICAM-1 values of our patients in each group were far fewer than those values in the previous paper namely those of survivors. This fact suggests that although there was significant difference in plasma ICAM-1 in two groups in our study, the extent of the increase in ICAM-1 may not have clinical or biological significance. However we have not proven this and slightly elevated plasma ICAM-1 in the TV6 group may be the cause of lower P/F ratio after the surgery. Lastly, postoperative oxygenation difference was the only clinical outcome between the groups. No patient experienced hypoxia postoperatively because each patient received supplemental oxygen at the PACU. However, the data suggest some patients, especially those in the TV6 group (mean P/F ratio of about 300), may have experienced hypoxia unless supplemental oxygen was applied. We may consider this as clinically significant.

## Conclusion

In conclusion, V_T_ of 6 mL•kg^−1^ predicted body weight ventilation with a PEEP of 3 cmH_2_O during hepatectomy caused inflammation in the airway and reduced oxygenation after the surgery, whereas V_T_ of 12 mL•kg^−1^ ventilation with a PEEP of 3 cmH_2_O did not. There appears to be more lung inflammation with low tidal volume with low PEEP, which may be due to repeated alveolar collapse and re-expansion (i.e., atelectrauma). Our study supports the findings of other investigations looking at lung protective ventilation during surgery, mainly that low PEEP levels may be harmful. Careful consideration is warranted when enforcing a lung-protective strategy during major surgery.

## Abbreviations

ANOVA, analysis of variance; ARDS, acute respiratory distress syndrome; BAL, broncho-alveolar lavage; BGA, blood gas analysis; BMS, bronchoscopic microsampling; DF, dilution factor; ELF, epithelial lining fluid; ELISA, enzyme-linked immunosorbent assay; F_I_O_2_, inspired oxygen fraction; I/E, the ratio of the duration of inspiration to the duration of expiration; ICAM-1, intercellular adhesion molecule-1; IL, interleukin; PaCO_2_, arterial partial carbon dioxide pressure; PACU, post-anesthetic care unit; PaO_2_, arterial partial oxygen pressure; PEEP, positive end-expiratory pressure; PMN, polymorphonuculear cell; TNF, tumor necrosis factor; TV12, patients ventilated with a V_T_ of 12 mL per predicted body weight; TV6, patients ventilated with a V_T_ of 6 mL per predicted body weight; V_T_, tidal volume
